# Three-Dimensional Lower Extremity Joint Loading in a Carved Ski and Snowboard Turn: A Pilot Study

**DOI:** 10.1155/2014/340272

**Published:** 2014-09-15

**Authors:** Miriam Klous, Erich Müller, Hermann Schwameder

**Affiliations:** ^1^Department of Health and Human Performance, College of Charleston, Charleston, SC 29424, USA; ^2^Department of Sport Science and Kinesiology, Christian Doppler Laboratory “Biomechanics in Skiing”, University of Salzburg, 5400 Hallein, Salzburg, Austria

## Abstract

A large number of injuries to the lower extremity occur in skiing and snowboarding. Due to the difficulty of collecting 3D kinematic and kinetic data with high accuracy, a possible relationship between injury statistic and joint loading has not been studied. Therefore, the purpose of the current study was to compare ankle and knee joint loading at the steering leg between carved ski and snowboard turns. Kinetic data were collected using mobile force plates mounted under the toe and heel part of the binding on skies or snowboard (KISTLER). Kinematic data were collected with five synchronized, panning, tilting, and zooming cameras. An extended version of the Yeadon model was applied to calculate inertial properties of the segments. Ankle and knee joint forces and moments were calculated using inverse dynamic analysis. Results showed higher forces along the longitudinal axis in skiing and similar forces for skiing and snowboarding in anterior-posterior and mediolateral direction. Joint moments were consistently greater during a snowboard turn, but more fluctuations were observed in skiing. Hence, when comparing joint loading between carved ski and snowboard turns, one should differentiate between forces and moments, including the direction of forces and moments and the turn phase.

## 1. Introduction

Skiing and snowboarding are the prominent winter sports and the general trend shows an increasing number of people participating in these sports [1–3]. With the increased number of practitioners, the number of injuries increased. Injury statistics have shown that skiing injuries mainly involve the lower extremities, predominantly the knee (18.1%–36.7%) [4–8] and the ankle joint (6%–12.2%) [6, 9–11]. In snowboarding, injuries occur when falling or during landings after a jump and mainly the upper extremities are injured [7, 8, 12]. However, still a considerable number of injuries occur in the lower extremities, with 6.4–17% in the knee joint and 4.9%–16% in the ankle joint [7, 13–16]. These values clearly show the vulnerability of the lower extremities in skiing and snowboarding. If we assume that higher joint loading is related to injuries, injury statistic would suggest greater knee joint loading in skiing and greater ankle joint loading in snowboarding.

It has been suggested that the introduction of the carved turning technique is contributing to the increase in the severity of lower extremity injuries in skiing. Based on biomechanical concepts described by Howe [17], external forces acting on skier/snowboarder include gravity and normal force, snow friction, air resistance, propulsion force, and, while turning, centripetal force. The characteristically higher velocity and smaller turn radius in carved turns increase the centripetal force and thereby increase the lower extremity joint loading. This concept applies to both skiing and snowboarding. However, the magnitude and direction of joint loading for each of the joints could vary between skiing and snowboarding due to technique, position, and equipment differences. With the use of soft boots in snowboarding, a minimal amount of movement in the ankle is expected, whereas in the stiff ski boots forces and moments are transferred to the knee joint. This would suggest higher joint loading in the ankle joint in snowboarding and higher joint loading in the knee joint in skiing and would be in agreement with the injury statistics described previously.

In a first attempt, it is of special interest to obtain greater insight into the differences in ankle and knee joint loading between a carved ski and snowboard turn. The focus of the current study was on a carved turn since a carved turn is a common skill in both skiing and snowboarding and higher joint loadings are predicted in this kind of turn. A study of Urabe et al. [18] on skiing reported larger number of injuries at the outer leg. The outer leg might experience higher forces and moments due to its steering function. Therefore, the current study focused on the steering leg. In snowboarding steering is controlled by the rear leg.

Several biomechanical studies estimated the joint loading in skiing while turning [19–24] and on landing maneuvers after a jump [25, 26]. Also in snowboarding forces and moments have been estimated, at the boot sole with an alpine board [27] and in lower extremity joints [28]. Besides the studies by Klous et al. [23] and Krüger et al. [28], none of the previous studies performed full three-dimensional (3D) inverse dynamic analysis in skiing or snowboarding with sufficient accuracy. This is due to the complexity to collect 3D kinematic data accurately in a field experiment [20]. Recently, we developed a method to collect accurate 3D kinematic data Klous et al. [29]. Comprehensive accuracy examination of the kinematic setup, kinematic data collection, and analysis led to photogrammetric errors of 11, 9, and 13 mm in *x*-, *y*-, and *z*-direction, respectively. The maximum error caused by skin movement artifacts was 39 mm; similar errors have been reported in laboratory settings [30]. Together with the collected 3D kinetic data, the kinematic data served as input for inverse dynamic analysis to determine lower extremity joint loading in full 3D with sufficient accuracy.

Therefore, the main purpose of the current study was to compare three-dimensional (3D) ankle and knee joint loading between carved ski and snowboard turns in the steering leg in a real life situation with high accuracy. Based on the injury statistics and due to differences in technique, position, and equipment (hard boot versus soft boot) between skiing and snowboarding, it was hypothesized that, at the steering leg in a carved turn, ankle joint loading was greater in snowboarding and knee joint loading was greater in skiing.

## 2. Methods

### 2.1. Subjects and Equipment

Five male skilled subjects participated in the experiment, three skiers (height: 174 ± 5.6 cm, weight: 75 ± 3.5 kg) on an all-round carver (length: 170 cm, side cut: 34 mm, ski radius: 17 m) and two regular snowboarders (height: 178 ± 2.8 cm, weight: 66.5 ± 4.9 kg) on a freestyle board (length: 158 cm, binding alignment: 25° front, 10° rear binding, distance between bindings: 53 cm). Subjects were ski and snowboard teachers at national level in Austria and had no history of injuries. Subjects were wearing their own ski/snowboard boots. All subjects gave their informed consent.

### 2.2. Kinematic Setup

**Figure 1:**
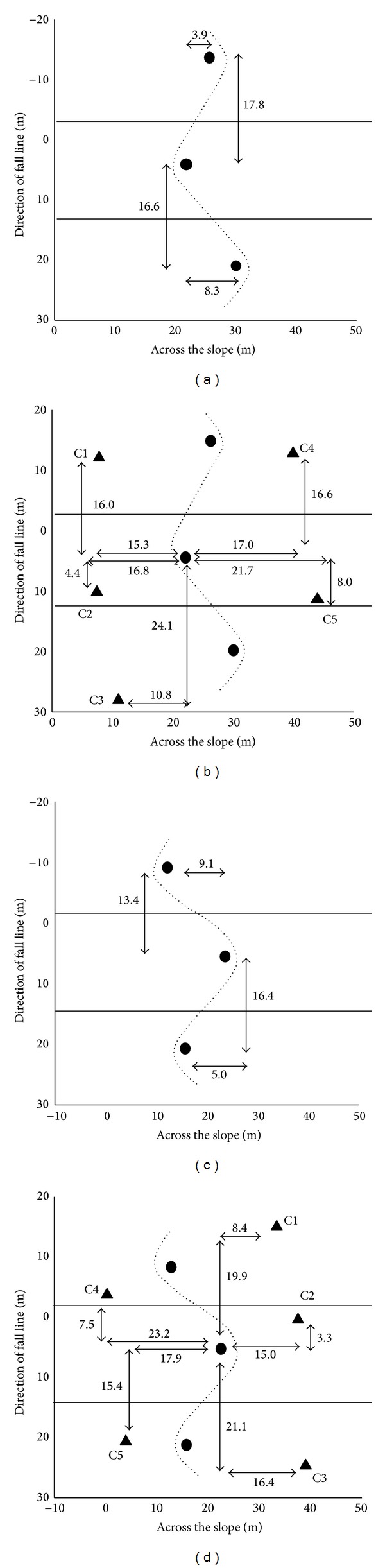
Course definition (a and c) and camera setup (b and d) for the ski turn (a and b) and snowboard turn (c and d) including gates (●), cameras (▲), and the part of the turn that is analyzed (in between the thick lines).

A detailed description of the kinematic setup can be found in Klous et al. [29]. A schematic representation of the kinematic setup is shown in Figure 1, including the course definition ((a) and (c)) and camera setup ((b) and (d)) for the ski turn ((a) and (b)) and snowboard turn ((c) and (d)). Briefly, the course was set with five gates and data were collected around the third gate. Slope inclination was 21° in skiing and 23° in snowboarding. Kinematic data were collected from edge change to the subsequent edge change (Figure 1, thick horizontal lines) with five synchronized panning, tilting, and zooming cameras (Panasonic, F15, 50 Hz).

A reference point system was set up on the hill to describe the 3D movement of the skier and snowboarder from two-dimensional (2D) video data using panning, tilting, and zooming cameras [29, 31, 32]. The positions of the camera tripods, the reference points, and the positions of the gates were measured using a theodolite. The kinematic setup allowed only one trajectory for skiing and one for snowboarding. Hence, the radii of the ski and snowboard turn were similar, but therefore the velocity of the turns varied. Approximately 100 markers were attached to a tight fitting stretch-suit on the pelvis, legs, ski/snowboard boots, and skies/snowboard. This procedure was necessary to have at least three markers per segment in sight of two successive cameras during the entire run which was required to perform 3D kinematic analysis [33].

### 2.3. Kinetic Setup

Stricker et al. [34] described in detail the kinetic setup including a thorough analysis of the accuracy of the system. Briefly, kinetic data was collected with a mobile force plate system (KISTLER, CH, 200 Hz) consisting of 4 six-component dynamometers that were mounted on the ski (two on each ski) or snowboard. The measurement error of the dynamometers was 0.3% for 3D forces (*F* > 292 N) and ranged from 4.0% to 8.3% for 3D torques. The deviation of the calculated point of force application from its reference was 1.4 and 8.8 mm in mediolateral and anteroposterior direction, respectively. Temperature had little impact on the measurement accuracy of the dynamometers [34]. The standing height from the snow to the bottom of the ski boot was 8 cm. Four cables connected the dynamometers with the charging amplifiers in a backpack that also contained the data loggers. The additional weight of the complete measuring device was approximately 7 kg.

### 2.4. Protocol

Prior to the experiment three test runs were performed for warm-up and adjustment of measurement devices. Additionally, subjects performed quiet stance trials parallel and orthogonal to the fall line to allow definition of local coordinate systems (LCSs) for each segment. Data were collected for a carved left turn in skiing and a carved front side (right) turn in snowboarding. For both skiing and snowboarding, three runs/trials were collected in which the subject was clearly visible in all videos and the technique was performed correctly (controlled by visual inspection). To allow synchronization of the kinetic and kinematic measuring devices in the data analysis, the subject performed a jump directly after the trial that was filmed by at least one camera. A second reset of the kinetic measuring device was performed after the run to control for possible drift behaviors of the system.

### 2.5. Data Analysis

Kinematic and kinetic data analyses as well as inverse dynamic calculations are described in detail in Klous et al. [29]. Briefly, 3D marker coordinates were calculated from two successive cameras after manually digitizing all visible markers for each video frame for each camera using SIMI Motion (Version 7.0, Build 242). Data were filtered and interpolated and the position and orientation of the segments were calculated using Cardan angles with mediolateral (*x*), posterior-anterior (*y*), and vertical (*z*) rotation sequence [35, 36] with software developed in Matlab (Version 6.5). Joint center positions were calculated using the sphere-fitting SCoRE method [37]. Kinetic data of the left and right leg were synchronized and offset corrected and kinematic and kinetic data were also synchronized.

Inertial properties of the lower extremities were calculated applying the geometric model by Yeadon [38]. The model was extended by adding ski/snowboard boots to the model. The parts of the boot below the ankle were added to the foot segment and the parts above the ankle were added to the shank segment. Density values from Dempster [39] were taken according to Yeadon [38] to calculate the inertial parameters of the segments. The experimentally determined densities for the inside and outside ski boot were 280 kg/m^3^ and 1400 kg/m^3^, respectively. The experimentally determined densities for the inside snowboard boot were 200 kg/m^3^ and for the outside boot 470 kg/m^3^.

**Figure 2:**
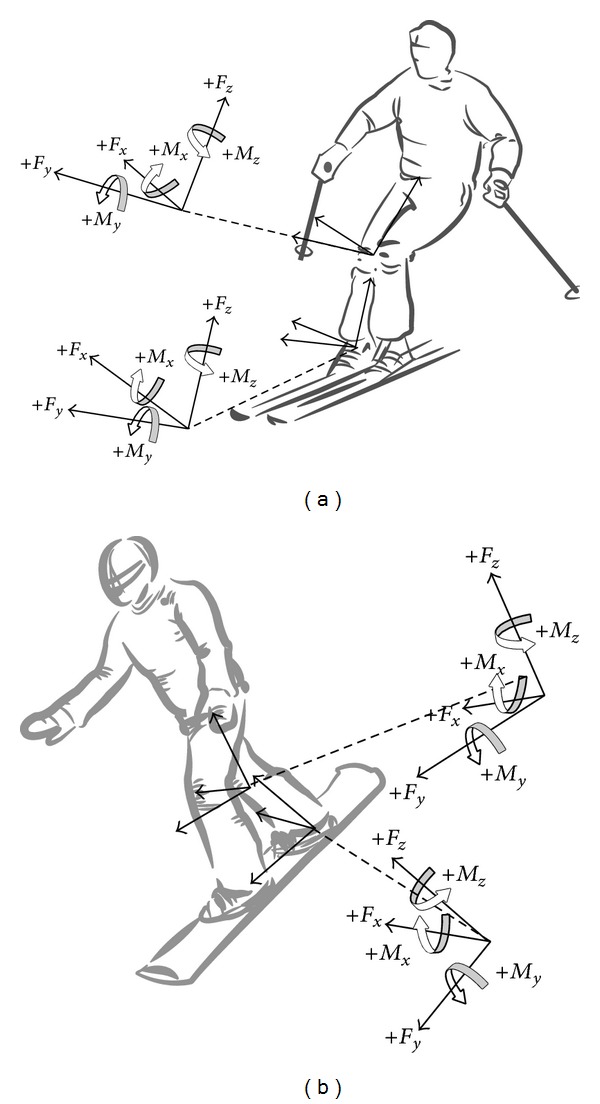
Definition of the local coordinate system (LCS) at the leg and the thigh of the steering leg in skiing (a) and snowboarding (b).

Inverse dynamics analysis was applied to calculate net joint forces and moments (net joint loading) from edge changing to the subsequent one. Since high frequencies kinematic movements were not expected, the global position of the center of mass (COM) as well as the orientation of each of the segments was filtered using a 4th order zero-phase Butterworth low-pass filter with a cutoff frequency of 2 Hz. Kinematic angular and linear acceleration data were determined by numerical differentiation and kinetic and kinematic data were time-normalized to arbitrarily chosen 201 data points before entering into the inverse dynamic analysis. Net joint forces and net moments at the ankle joint and knee joint were calculated in the LCS of the calf and thigh, respectively (Figure 2). Net joint forces were normalized to body weight (BW) and net joint moments were normalized to body mass. The normalized net forces and net moments (referred to as joint forces and joint moments throughout the remaining paper) at the ankle joint represented the net forces and net moments acting from the foot at the leg calculated in the LCS of the leg. The net forces and net moments at the knee joint represented the net forces and net moments acting from the leg at the thigh calculated in the LCS of the thigh. The LCSs were defined with the *y*-axis in anterior-posterior direction (positive *y*-axis anterior), the *z*-axis along the length of the segment (positive *z*-axis proximal), and the *x*-axis in mediolateral direction, with the positive *x*-axis pointing lateral for the steering (right) leg in both skiing and snowboarding (Figure 2).

Due to the complexity of the experimental setup and the related difficulty to collect accurate data, only in two trials a limited amount of interpolation was necessary to fulfill the requirement of three markers in sight of two successive cameras during the entire run. Therefore, in the following, one representative carved ski turn and one representative carved snowboard turn are presented comparatively. Ankle and knee joint loading in the steering leg in skiing (outside leg) and snowboarding (rear leg) were compared in the current study. Data were divided into three phases of equal duration (33%). These phases correspond approximately to the functional aspects of the turn: initiation phase, steering phase I, and steering phase II [40, 41].

A skidding angle *β* was calculated describing the skidding component in a turn [42, 43]. This angle was defined as the angle between the orientation vector (line from the front to the rear binding piece of the ski) and the velocity vector of the ankle of the skier/snowboarder's leg. In the current study an average skidding angle was calculated for skiing by averaging the positions of the rear-binding piece of both skies, the positions of the front binding piece of both skies, and the ankle joint position of the right and left leg. In snowboarding, an average ankle joint position was calculated. With the angle *β* can objectively be verified that turns were carved. Before calculating the skidding angle, position data were filtered with a 5 Hz low-pass 4th order, zero-lag Butterworth filter [23, 42].

Since only one trial for each discipline is compared only descriptive statistics are reported with means and standard deviations for each of the three phases of the turn.

## 3. Results

### 3.1. Turning Technique

**Figure 3:**
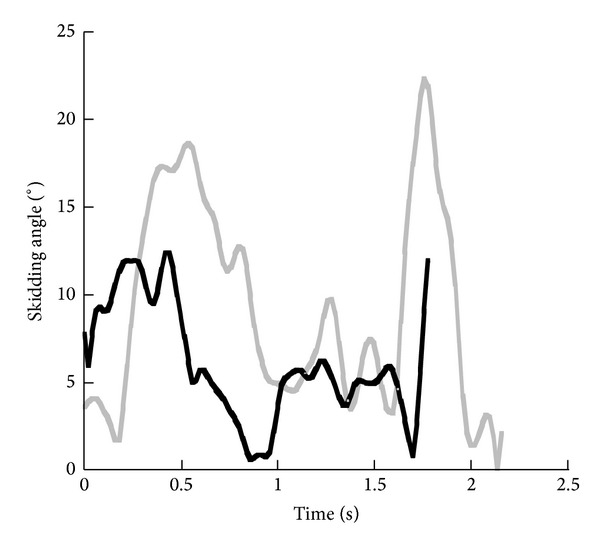
Average skidding angle *β* in a ski turn (black) and a front side snowboard turn (grey).

A skidding angle *β* was calculated to verify the proper performance of the turning techniques (Figure 3). The average angle in skiing was 6.1° (±3.2°) and in snowboarding 9.2° (±5.9°). The average velocity was 13.9 m/s and 11.1 m/s in skiing and snowboarding, respectively. The maximum velocity in skiing was 16.5 m/s and in snowboarding 11.9 m/s. Note that the ski and snowboard turn were performed with similar turning radii, but different velocities.

### 3.2. Ankle Joint Loading at the Steering Leg

**Figure 4:**
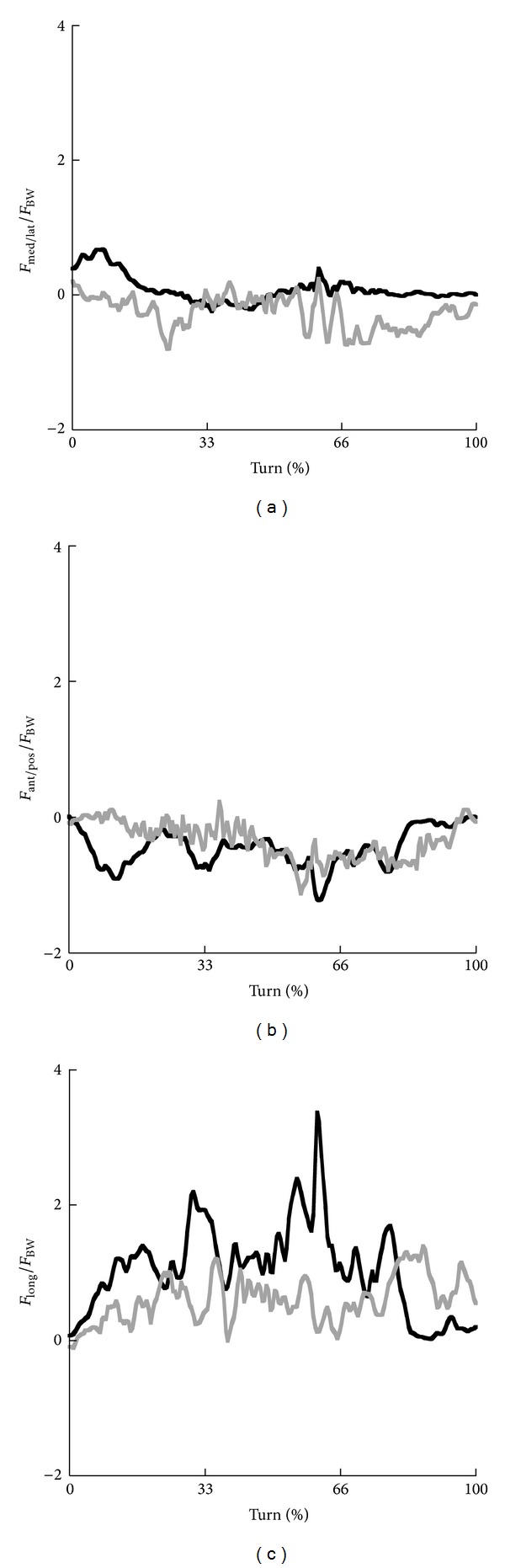
Time profiles of the net medial (−)/lateral (+) forces (a), net anterior (+)/posterior (−) forces (b), and net forces around the longitudinal axis (c) at the ankle joint for the steering leg in skiing (black) and snowboarding (grey).

Time profiles of the mediolateral forces, anterior/posterior forces, and longitudinal forces at the ankle joint in skiing and snowboarding are shown in Figure 4 and Table 1. Mediolateral forces and anterior/posterior forces were clearly lower than the forces along the longitudinal axis. In both skiing and snowboarding, ankle joint forces acted in posterior and upward direction. Longitudinal forces in skiing were higher than in snowboarding. These forces increase up to 2-3 times BW at 60% of the turn in skiing, whereas in snowboarding the longitudinal force was rather consistent at approximately 1·BW. Smaller forces in posterior direction showed more variation in skiing than in snowboarding. Average ankle joint forces in mediolateral were rather similar for skiing and snowboarding in the first two phases, but higher in snowboarding in the last phase. The ankle joint forces in anterior/posterior direction were similar for the last two phases, but in the first phase the anterior/posterior force was higher in skiing. The longitudinal forces were clearly greater in skiing than in snowboarding in the first two phases and higher in longitudinal direction than in the other directions. In snowboarding the longitudinal force was more consistent throughout the phases.

**Figure 5:**
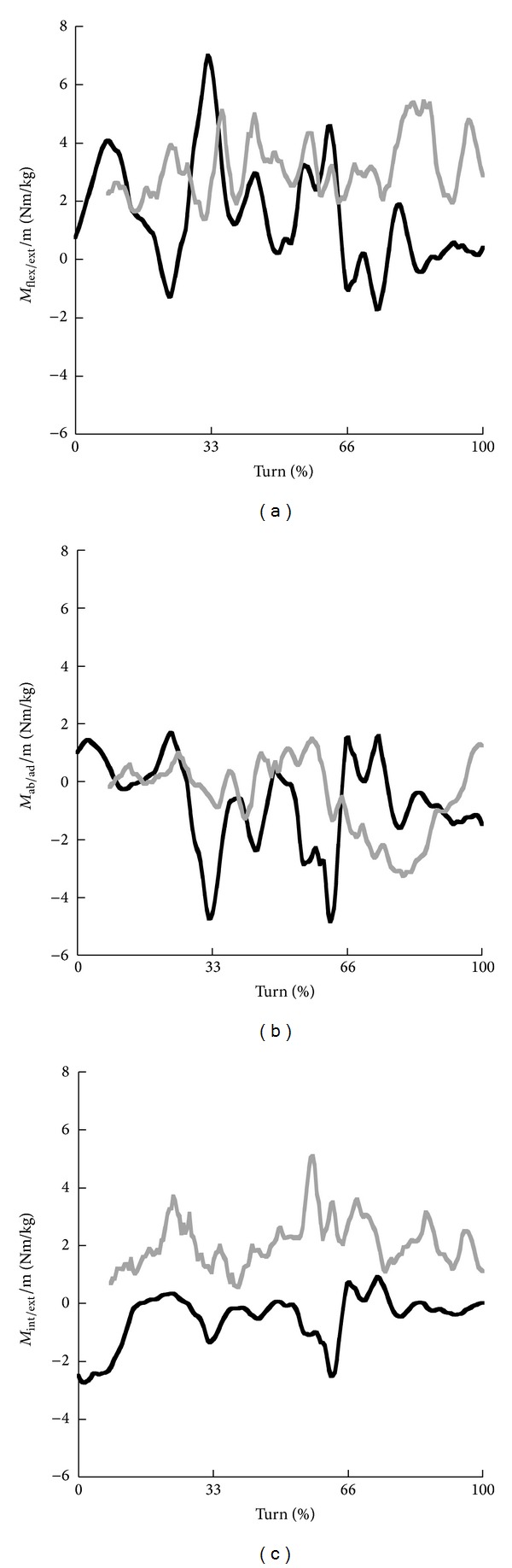
Time profiles of the net flexion (+)/extension (−) moments (a), net adduction (+)/abduction (−) moments (b), and net internal (+)/external (−) moments (c) at the ankle joint for the steering leg in skiing (black) and snowboarding (grey).

During the turn predominantly an extension moment and abduction moment acted at the ankle joint in both skiing and snowboarding (Figure 5). Furthermore, an internal rotation moment acted at the ankle joint in snowboarding and an external rotation moment in skiing. Time profiles of flexion/extension moments showed more variations in skiing than in snowboarding with fluctuations between −1 and 7 Nm/kg, whereas the extension moment in snowboarding varied between 2 and 5 Nm/kg. Average magnitudes (Table 2) showed higher flexion/extension moments in snowboarding but larger fluctuations in skiing in the first and second phase of the turn represented by the large standard deviation (SD). A large abduction moment in skiing was observed in the second phase with peak values over 4 Nm/kg and an average value of 1.7 Nm/kg. In snowboarding the abduction moment was approximately 0 Nm/kg in the first and second phases (see also Table 2) but increased up to 3 Nm/kg and averaged 1.6 Nm/kg in the third phase. The internal rotation moment clearly showed larger average magnitudes in all three phases in snowboarding than in skiing (Table 2).

### 3.3. Knee Joint Loading Steering Leg

**Figure 6:**
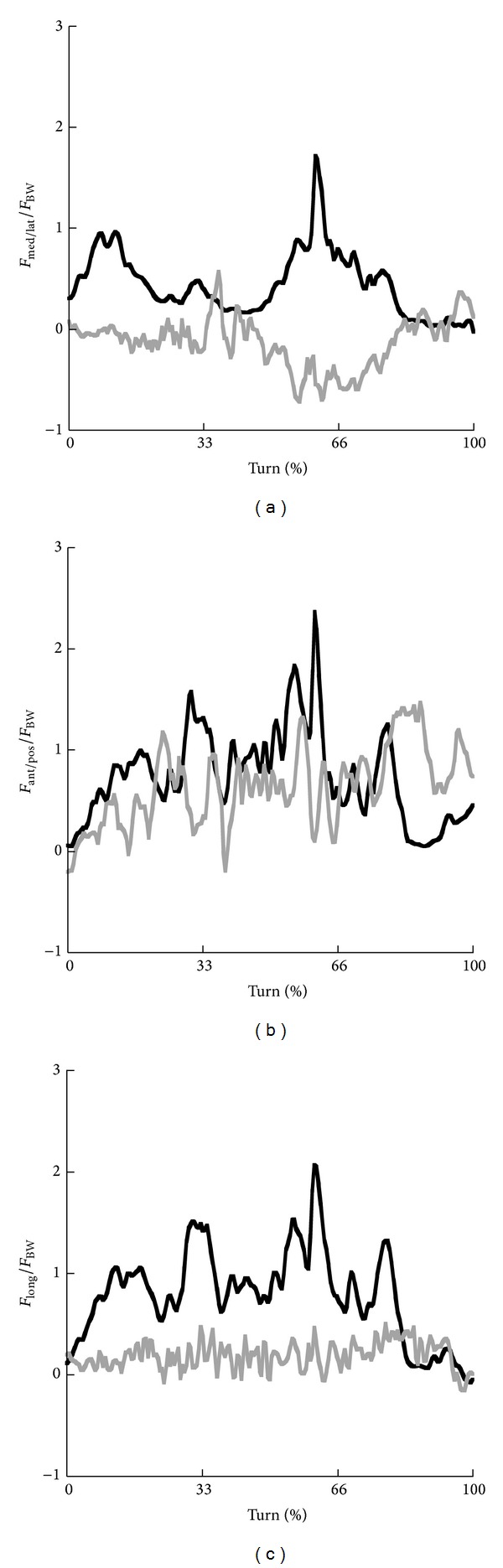
Time profiles of the net medial/lateral forces (a), net anterior/posterior forces (b), and net forces around the longitudinal axis (c) at the knee joint for the steering leg in skiing (black) and snowboarding (grey).

Similar time profiles were observed for the forces in anterior/posterior direction for skiing and snowboarding till approximately 70% of the turn with slightly lower values in snowboarding (Figure 6). In the third part of the turn, the force in the anterior direction is clearly higher in snowboarding than in skiing. This is confirmed by the average magnitudes for each of the three phases presented in Table 3. Anterior/posterior forces and forces along the longitudinal axis of the knee joint showed similar patterns in skiing. Until 60% of the turn, forces increased up to approximately 2·BW and then decreased. Longitudinal forces in snowboarding varied around 0·BW. Forces in medial/lateral direction showed opposite time profiles at the steering leg for skiing and snowboarding. The lateral force in skiing showed a larger increase between 50 and 75% of the turn and a smaller increase in the first 25% of the turn. In snowboarding, this increase was only observed between 50 and 75% of the turn in medial direction. Average magnitudes for medial/lateral forces for all three phases were larger in skiing than in snowboarding.

**Figure 7:**
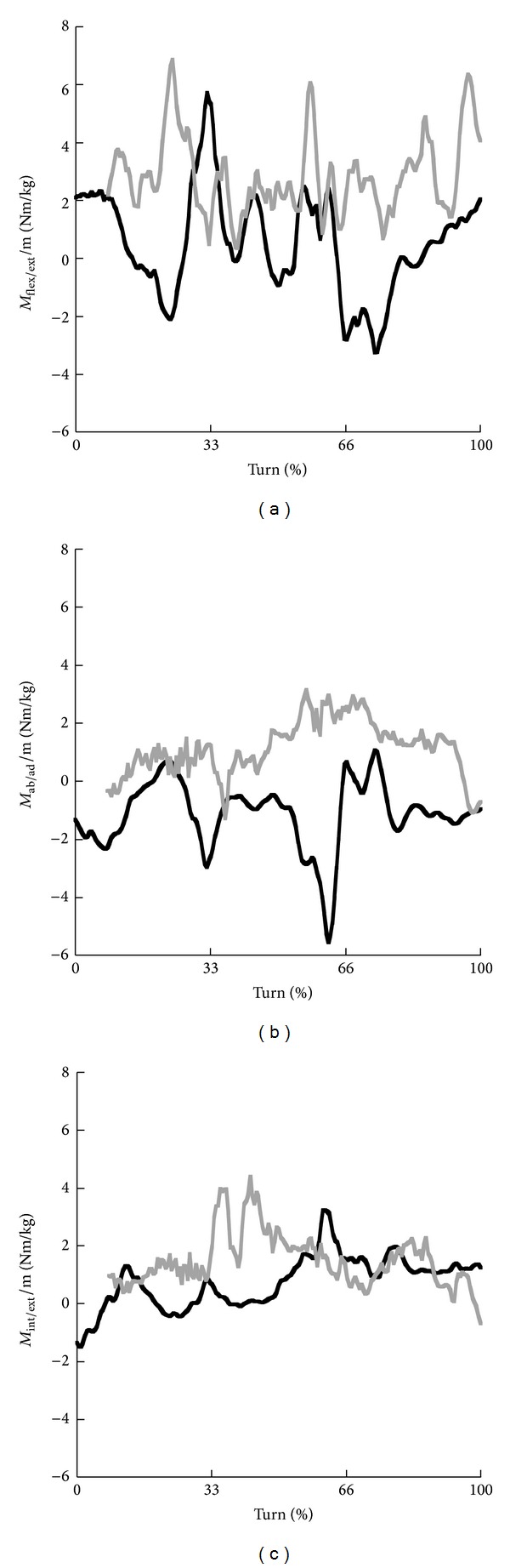
Time profiles of the net flexion (+)/extension (−) moments (a), net adduction (+)/abduction (−) moments (b), and net internal (+)/external (−) moments (c) at the knee joint for the steering leg in skiing (black) and snowboarding (grey).

The time profiles of the moments at the knee joint were rather different for skiing and snowboarding (Figure 7). In skiing the moment varied between flexion and extension throughout the turn with magnitudes between approximately −2 and 4 Nm/kg. In snowboarding, a flexion moment acted at the knee joint throughout the turn with magnitudes up to 6 Nm/kg. Average magnitudes were clearly higher in snowboarding for all three phases, but the larger SD in skiing for all three phases represented the larger fluctuations in skiing (Table 4). Furthermore, in skiing an abduction moment acted at the knee joint, whereas in snowboarding an adduction moment throughout the turn. Average magnitudes were clearly larger in skiing in phase 1 and in snowboarding in phase 3. In phase 2 average magnitudes were approximately similar, but in opposite directions (Table 4). Rather similar time profiles were observed for the internal/external rotation moment at the knee joint. Both in skiing and snowboarding acted an internal rotation moment during most of the turn. However, average magnitudes were clearly higher in snowboarding than in skiing in the first and second phases of the turn. In the third phase these magnitudes were similar (see Table 4).

## 4. Discussion

The aim of this study was to compare the ankle and knee joint loading at the steering leg between a carved ski and snowboard turn. Based on reported injury statistics and due to differences in technique, position, and equipment between skiing and snowboarding, it was hypothesized that ankle joint loading was greater in snowboarding and knee joint loading was greater in skiing. However, the current study showed a different outcome. While forces were mostly similar for skiing and snowboarding, the joint moments were consistently greater during a snowboard turn, whereas in skiing much more fluctuations were observed during the turn, particularly in the first and second phase of the turn (represented by the greater standard deviation in skiing in those two phases). Moreover, forces along the longitudinal axis were higher in skiing than in snowboarding.

Results showed that the carved turn demonstrated some skidding components. The average skidding angle calculated across time was higher in snowboarding than in skiing, which could be due to the rather steep slope to perform a carved turn in snowboarding. Nevertheless, both turns were representative of a carved turn. Results were in agreement with Müller et al. [43] and Wagner [42] who reported average skidding angles for the carving technique in skiing of 4.1°. Knünz et al. [44] reported angles in a carved ski turn of 1-2° for the outer leg and 7-8° for the inner leg in a (purely) carved ski turn.

Forces in anterior/posterior and medial/lateral direction at the ankle joint were similar and rather low for skiing and snowboarding. As a consequence it is expected that the internal/external rotation moment is also rather low as is observed in skiing. However, in snowboarding internal rotation moments reached magnitudes of approximately 2 Nm/kg. Consistent and larger values throughout the turn were also observed for the flexion/extension moment in snowboarding, whereas the force along the longitudinal axis was below 1·BW and the anterior/posterior force was even lower. Krüger et al. [28] reported even larger peak values for the flexion/extension moment at the ankle joint compared to the current study but do not report if these values are a consequence of large kinetic or kinematic values. With the low forces observed in the current study, these relatively high moments must be due to kinematics, hence angular accelerations of the segments, or due to the different body positions in skiing and snowboarding which is represented by the position of the joint centres with respect to the force vector. The use of soft boots in snowboarding allowed short but fast rotational movements (i.e., kinematic parameters), whereas these movements were not possible with stiff ski boots. These equipment differences would explain the greater joint moments at the ankle joint in snowboarding. This was supported by a study of Delorme et al. [45] that compared ankle joint kinematics between stiff and soft boots in snowboarding. This study reported that the use of soft boots leads to larger average dorsi/plantar flexion angles and internal/external rotation angles, as well as larger maximum dorsi/plantar flexion angles, eversion/inversion angles, and internal/external rotation angles, larger minimal internal/external rotation angles, and a larger range of motion in dorsi/plantar flexion.

In skiing, the time pattern of the force along the longitudinal axis at the ankle joint showed similarities with the time pattern of the flexion/extension and abduction/adduction moments, but in opposite direction. Hence, opposite to snowboarding, the large moments in skiing seemed to be a consequence of the produced forces. Note that, in skiing, the flexion/extension moment allowed the movement to the tip/tail of the ski, whereas the abduction/adduction moment places the ski at the edges (see Figure 2). Fluctuations (represented by the standard deviation) were much larger for the moments than for the forces and also much larger in skiing than in snowboarding. This might suggest that the greater number of injuries at the ankle joint is caused by the specific body position in snowboarding and the consistently high moments due to kinematic variables, rather than large fluctuation as observed in the moments in skiing.

At the knee joint both medial/lateral forces and forces along the longitudinal axis were higher in skiing, whereas the anterior/posterior forces were similar for skiing and snowboarding. However, the higher forces in skiing did not result in consistently higher moments compared to snowboarding. The flexion/extension moments in snowboarding were required to place the snowboard at the edges, just like the abduction/adduction moment in skiing. The flexion/extension moments in snowboarding were approximately 3 Nm/kg, whereas the abduction/adduction moments in skiing were approximately 1.0–1.5 Nm/kg. Also the flexion/extension moments in skiing were approximately 1 Nm/kg as were the abduction/adduction moments in snowboarding. In general, moments were slightly lower at the knee joint than at the ankle joint in snowboarding, whereas in skiing the opposite was observed. Again, the larger moments in snowboarding seemed not to be due to the high forces but due to the soft boot allowing larger accelerations and a different body position in snowboarding than in skiing.

Even though the fluctuations were larger in snowboarding at the knee than at the ankle joint, these variations were still much lower in snowboarding than in skiing. These fluctuations represent the loading and unloading that are clearly greater in skiing than in snowboarding. In situations when a skier has to make a sudden adjustment, these peak values would increase even further. In skiing, joint moments increased in the knee joint compared to the ankle joint, whereas in snowboarding the moments decreased. Besides the knee joint forces being similar or greater in skiing than in snowboarding, also the peak forces and moments were larger in skiing than in snowboarding, except for the internal/external rotation moment. Krüger et al. [28] reported clearly lower peak values for the flexion/extension moment in snowboarding (33% less) than in the current study, which would make differences between skiing and snowboarding even more pronounced. These three aspects together could be an explanation for the larger amount of knee injuries in skiing than in snowboarding.

Even though the joint loading observed in the current study is rather high, one should realise that many other aspects can explain the injury statistics as presented in the current study. The quality of the snow, the technical and physical capability of the skier or snowboarder, and the large number of skiers and snowboarders at the slope could explain the many injuries that occur in skiing and snowboarding. The skier and snowboarder in the current study carried additional equipment to allow measurement of ground reaction forces. This equipment influenced their weight and their standing height. With their level of expertise, the skier and snowboarder did not report any influence of this equipment. Nevertheless, the equipment might have influenced their technique and performance. Additionally, the differences in stiffness between ski and snowboard boots could have influenced the results. Due to the stiff ski boot, part of the loading might have been transferred to the boot and thereby reduced the ankle joint in skiing. Inverse dynamic calculations did not allow determining how much of the ankle joint was transferred to the ski boot. Hence, this could have caused overestimation of the ankle joint in skiing. However, where the current results showed larger ankle joint in snowboarding, the difference in ankle joint between skiing and snowboarding would have even been greater if the ankle joint in skiing was overestimated. When current results showed larger ankle joint in skiing, these differences might not have been as profound. Both situations support the research hypothesis. Also, the magnitudes of the ankle joint forces and moments in skiing might have been lower, but it is not to expect that the time patterns were influenced. Furthermore, the kinematic setup allowed a ski and snowboard turn to be performed with similar radii but different velocities. The centripetal force (*F*_*c*_) in a turn is influenced by the velocity (*F*_*c*_ = *mv* ^2^/*r*). Although the velocity in snowboarding was lower than in skiing, the ankle and knee joint forces and moments were not consistently lower than in skiing. We speculate that if the snowboard turn was performed with higher velocities, the forces and moments at the ankle and knee joint would further increase due to an increase of the centripetal force. Furthermore, videos and data of ground reaction forces throughout the collected data were similar. Nevertheless, the findings should be interpreted with caution due to the single subject design. Additionally, even though the applied method shows a good accuracy for on-snow data collection, the results of inverse dynamic calculations depend strongly on the accuracy of the input data. As is shown by McCaw & DeVita [46] errors in the input data are propagated in the inverse dynamics procedures, thereby reducing the accuracy of the results calculated using this procedure. Finally, it is important to emphasise that we calculated forces and moments during successful turns, which are not representative of the forces and moments during unsuccessful turns that result in falling and/or injury.

## 5. Conclusion

The expected higher ankle joint loading in snowboarding and higher knee joint loading in skiing that was based on reported injury statistics in the lower extremities in skiing and snowboarding and the differences in position, technique, and equipment (soft boot versus hard boot) could not be confirmed. Ankle joint loading was not consistently greater in snowboarding than in skiing and vice versa for the knee joint loading. When comparing skiing and snowboarding, differentiation was required between forces and moments, the direction of the forces and moments, and the phase of the turn that was considered. However, there seemed to be a trend that forces were larger in skiing and moments showed large fluctuations (loading-unloading), whereas in snowboarding high moments with a more consistent pattern were observed. In future research it is important to increase the number of participants in the study and study joint loading of various turning techniques.

## Figures and Tables

**Table 1:** Average net ankle joint forces in medial (−)/lateral (+) direction (*F*_med-lat_), anterior (+)/posterior (−) direction (*F*_ant-pos_), and along the longitudinal axis (*F*_long_) and standard deviations in the steering leg in skiing and snowboarding for each of the three phases.

	*F*_med-lat_/*F*_BW_ (SD)		*F*_ant-pos_/*F*_BW_ (SD)		*F*_long_/*F*_BW_ (SD)	
	Ski	Snowboard	Ski	Snowboard	Ski	Snowboard
Phase 1	0.2 (0.3)	−0.2 (0.2)	−0.5 (0.3)	−0.1 (0.1)	1.0 (0.5)	0.4 (0.3)
Phase 2	0.0 (0.1)	−0.1 (0.2)	−0.6 (0.2)	−0.5 (0.3)	1.5 (0.6)	0.6 (0.3)
Phase 3	0.0 (0.1)	−0.4 (0.2)	−0.3 (0.3)	−0.4 (0.2)	0.6 (0.5)	0.8 (0.3)

**Table 2:** Average net ankle joint flexion (−)/extension (+) moments (*M*_flex-ext_), net adduction (+)/abduction (−) moments (*M*_ad-ab_), and net internal (+)/external (−) rotation moments (*M*_int-ext_) and standard deviations in the steering leg in skiing and snowboarding for each of the three phases.

	*M*_flex-ext_/*m* (SD) (Nm/kg)		*M*_ad-ab_/*m* (SD) (Nm/kg)		*M*_int-ext_/*m* (SD) (Nm/kg)	
	Ski	Snowboard	Ski	Snowboard	Ski	Snowboard
Phase 1	2.2 (2.0)	2.5 (0.7)	0.0 (1.6)	0.2 (0.4)	−0.9 (1.1)	1.9 (0.8)
Phase 2	2.2 (1.5)	3.3 (0.8)	−1.7 (1.5)	0.1 (0.9)	−0.6 (0.7)	2.3 (1.0)
Phase 3	0.1 (0.8)	3.6 (1.1)	−0.5 (0.9)	−1.6 (1.3)	−0.0 (0.4)	2.1 (0.7)

**Table 3:** Average net knee joint forces in medial (−)/lateral (+) direction (*F*_med-lat_), anterior (+)/posterior (−) direction (*F*_ant-pos_), and along the longitudinal axis (*F*_long_) and standard deviations in the steering leg in skiing and snowboarding for each of the three phases.

	*F*_med-lat_/*F*_BW_ (SD)		*F*_ant-pos_/*F*_BW_ (SD)		*F*_long_/*F*_BW_ (SD)	
	Ski	Snowboard	Ski	Snowboard	Ski	Snowboard
Phase 1	0.5 (0.2)	−0.1 (0.1)	0.7 (0.4)	0.4 (0.3)	0.8 (0.3)	0.2 (0.1)
Phase 2	0.5 (0.4)	−0.2 (0.3)	1.1 (0.4)	0.6 (0.3)	1.1 (0.3)	0.2 (0.1)
Phase 3	0.3 (0.2)	−0.1 (0.3)	0.4 (0.3)	0.9 (0.3)	0.5 (0.4)	0.2 (0.2)

**Table 4:** Average net knee joint flexion (+)/extension (−) moments (*M*_flex-ext_), net adduction (+)/abduction (−) moments (*M*_ad-ab_), and net internal (+)/external (−) rotation moments (*M*_int-ext_) and standard deviations in the steering leg in skiing and snowboarding for each of the three phases.

	*M*_flex-ext_/*m* (SD) (Nm/kg)		*M*_ad-ab_/*m* (SD) (Nm/kg)		*M*_int-ext_/*m* (SD) (Nm/kg)	
	Ski	Snowboard	Ski	Snowboard	Ski	Snowboard
Phase 1	1.1 (2.0)	3.3 (1.4)	−1.0 (1.1)	0.6 (0.5)	0.0 (0.7)	1.0 (0.3)
Phase 2	0.8 (1.4)	2.3 (1.2)	−1.6 (1.4)	1.4 (1.0)	1.0 (1.0)	2.3 (0.9)
Phase 3	−0.4 (1.5)	3.0 (1.3)	−0.8 (0.7)	1.3 (1.0)	1.3 (0.3)	1.0 (0.7)
